# Glaucoma and dietary intake: a scoping review

**DOI:** 10.3389/fnut.2024.1497366

**Published:** 2024-12-10

**Authors:** Genesis Daniel Edokpa, Shelly Rose-Marie McFarlane

**Affiliations:** Caribbean Institute for Health Research, University of the West Indies, Kingston, Jamaica

**Keywords:** glaucoma, dietary intake, dietary habits, nutrition, scoping review

## Abstract

**Introduction:**

Studies have suggested that dietary intake may influence the incidence and progression of open-angle glaucoma. However, dietary modification is not usually included in the clinical management of glaucoma. The aim of this scoping review was therefore to map the evidence and determine the nature and extent of research done on “diet and glaucoma” and identify any gaps in this area of scholarship.

**Materials and methods:**

A comprehensive search of academic literature was conducted from two relevant electronic databases: PubMed and ScienceDirect. Primary studies that explored the relationship between dietary intake and glaucoma were included if the principal exposure was “diet” and if dietary habits were assessed with dietary questionnaires. The glaucoma outcomes of interest were visual field, retinal nerve fibre layer and/or optic nerve head features.

**Results:**

Nineteen studies were included in the final qualitative synthesis. The dates of publication ranged from 2003 to 2023. About 80% of the studies found some significant associations between glaucoma and dietary intake. However, most studies (95%) were observational, i.e., 7 (37%) used a cross-sectional design, 10 (53%) used a prospective cohort design; and 1 (or 5%) used a nested case–control study design. Only 1 study (or 5%) used a randomized intervention trial. Furthermore, while all studies investigated dietary intake with questionnaires, only 2 studies (or 11%) went further to include assessment of nutritional biomarkers.

**Conclusion:**

Although miscellaneous evidence supports the concept that diet may play a role in glaucoma, most data are unfortunately observational without proven causality, reporting associations from subjective dietary questionnaires. More well-designed studies are required, especially randomized controlled trials that can prove causality.

## 1 Introduction

Glaucoma is a progressive optic neuropathy characterized by degeneration of retinal ganglion cells which leads to visual field loss. It is a major cause of blindness worldwide (1–3). Clinically, glaucoma is divided into primary (open-angle and angle-closure glaucoma) and secondary (originating from trauma, medication, inflammation, cancer, or other conditions). The most common form of glaucoma is primary open-angle glaucoma (POAG).

Raised intraocular pressure (IOP) is the most critical and treatable risk factor for glaucoma, and the treatments currently available for managing it focus on lowering patients’ IOP (1–3). However, some types of glaucoma develop even within a normal IOP (1–3). This suggests that other independent factors might also influence the progression of glaucoma (3). Some other proposed mechanisms that have been reported include impaired blood flow, oxidative stress, excitotoxicity, and ocular rigidity (1–3). There are reports describing modifiable lifestyle risk factors for open-angle glaucoma such as heavy smoking and low consumption of certain fruits, vegetables and fatty fish (4).

Studies have shown that high intake of certain dietary components, including tea, fruits, and vegetables (especially green leafy vegetables) and vitamins A, B1, B2, and B3 may influence IOP and incidence of glaucoma (3, 5). This may be because they contain high concentrations of antioxidants, and flavonoids, and thereby have anti-inflammatory and neuroprotective properties (5). Despite this, dietary modification is not usually included in routine clinical management of glaucoma. Therefore, the main objective of this study was to conduct a scoping review to systematically map the evidence and extent of research done on diet and glaucoma and identify any existing gaps in literature.

A scoping review of primary studies on diet and glaucoma was carried out to delineate the range and nature of research that has been undertaken in this topic and identify any existing gaps in knowledge. “Primary study” here implies the methodology researchers use to collect data directly rather than depending on data collected from previously done research. The following research questions were formulated: What is the scope of evidence available on dietary intake and glaucoma? What is the extent, range and nature of research that has been undertaken on diet and glaucoma? What are the existing gaps in knowledge in this area?

## 2 Materials and methods

This review aligned with the Preferred Reporting Items for Systematic Reviews and Meta-Analyses extension for Scoping Reviews (PRISMA-ScR) and was based on an unpublished protocol (6).

### 2.1 Eligibility criteria

Published primary works that explored the relationship between dietary exposure and some glaucoma outcome in humans were included in this review. Studies were included if the primary exposure was “diet” or explicitly mentioned by the study author as “dietary” and if dietary habits were assessed with dietary questionnaires. The glaucoma outcomes of interest were visual field, retinal nerve fibre layer, and optic nerve head features such as cup-to-disc ratio.

All review articles, encyclopedias, book chapters, conference abstracts, book reviews, case reports, conference info, correspondence, data articles, discussion, editorials, errata, examinations, mini-reviews, news, patent reports, practice guidelines, product reviews, short communications and animal studies were excluded. Studies that had dietary supplements as primary exposure were also excluded.

### 2.2 Information sources and search strategy

A comprehensive search of academic literature was conducted from two relevant electronic databases: PubMed (1947 till present) and ScienceDirect (2001 till present), using the sole search term ‘diet and glaucoma’. Our first search was conducted on February 13, 2024, and the most recent search was executed on April 14, 2024.

### 2.3 Screening

The search results were first compiled, and duplicates were removed. For feasibility purposes, a single reviewer performed an initial screening of all unique records to identify those titles/abstracts that were clearly unrelated to the study objective (i.e., were not primary studies, did not have an exposure related to diet, and an outcome related to glaucoma). Following the initial screen, two independent reviewers completed a formal assessment for eligibility at the title/abstract level. The studies mutually agreed on for inclusion by both reviewers were then assessed at full-text level by one reviewer to confirm eligibility.

### 2.4 Data collection and analysis

Data extraction was completed for all included studies. Prior to beginning the extraction process, a comprehensive Microsoft Excel data extraction form was developed. The form consisted of information on authors, year of publication, country of origin where the study was conducted, study aims or purpose, research design, sample size, population age, validity/reliability of dietary questionnaires, how outcomes were measured, key findings and gaps in research. The results were presented qualitatively in various descriptions, frequency counts, and diagrams.

## 3 Results

**Figure 1:**
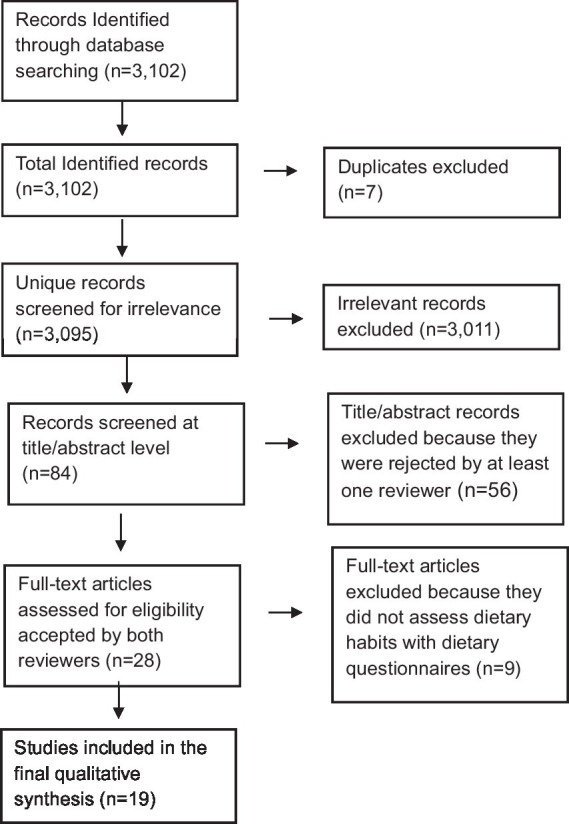
PRISMA diagram showing the study inclusion process.

The academic literature search returned 3,102 unique records, 3,011 of which were excluded through an initial screening for relevance (Figure 1). A total of 84 records were then screened for eligibility by two independent reviewers at the title/abstract level and 28 studies were mutually agreed on for inclusion by both reviewers. These were then assessed at full-text level. Nine studies were then excluded because they did not assess dietary intake and habits. Nineteen studies were afterwards included in the final qualitative synthesis.

**Figure 2:**
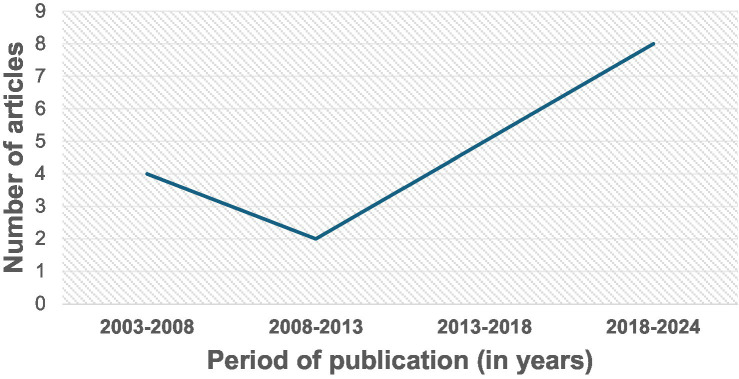
Dates of publication of original investigations on glaucoma and dietary intake. The dates ranged from 2003 to 2023, with most studies published in 2019 or later (42%, *n* = 8).

**Figure 3:**
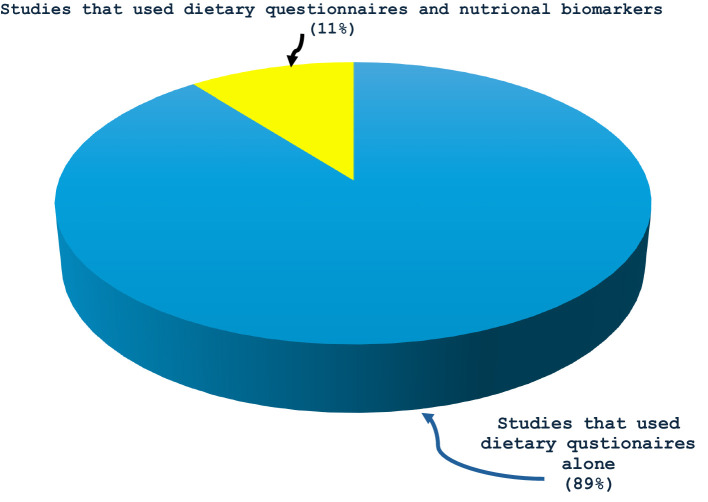
Proportions of primary investigations on glaucoma and dietary intake that used that used only dietary questionnaires to assess dietary intake (89%) and those that used both dietary questionnaires and nutritional biomarkers (11%).

**Figure 4:**
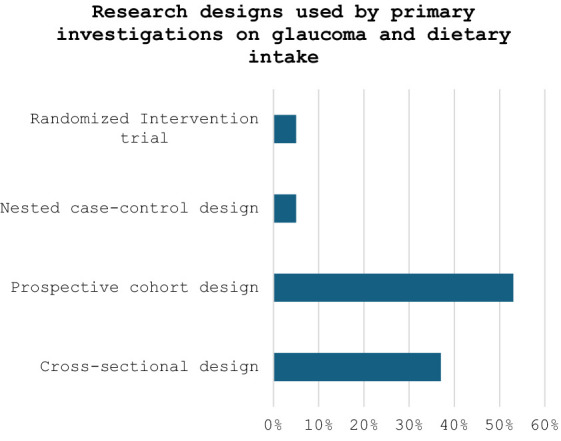
Percentages of observational and experimental studies used by primary investigations on glaucoma and dietary intake.

All the 19 studies were quantitative, and the dates of publication ranged from 2003 to 2023, with most studies published in 2019 or later (42%, *n* = 8) (Figure 2). They spanned seven different countries: USA (*n* = 9 studies or 47%), The Netherlands (*n* = 3 or 16%), Japan (*n* = 2 or 11%), Spain (*n* = 2 or 11%), Greece (*n* = 1 or 5%), Korea (*n* = 1 or 5%), and Poland (*n* = 1 or 5%). All the studies assessed dietary exposures and addressed potential confounders with questionnaires. 14 studies (74%) reported use of validated dietary questionnaires, and 5 studies (26%) did not indicate if their dietary assessment tool was validated (Table 1). No study gave information about the reliability of their dietary questionnaires. Semiquantitative food frequency questionnaires (FFQs) were mostly used to assess dietary intake. Other dietary questionnaires used were the block FFQs and the 24-h recall method. While all studies investigated dietary intake with questionnaires, 2 studies (or 11%) went further to include the investigation of nutritional biomarkers (Figure 3). Primary open-angle glaucoma (POAG) was the most common type of glaucoma investigated. The most common glaucoma outcomes were visual field indices (from visual field analysers), and optic nerve head features (from fundus photography). The observed optic nerve head features included: horizontal and/or vertical cup-to-disc ratio (CDR), appearance of optic disc hemorrhages, presence of retinal nerve fibre layer (RNFL) defects, violation of the ISNT rule (neuroretinal rim thickness in the order of inferior > superior > nasal > temporal), and CDR asymmetry. 2 out of 19 studies included RNFL defects along with optical coherence tomography (OCT) and visual fields in their investigations (3, 7). The sample sizes in the various studies ranged from *n* = 100 to *n* = 185,638 (8, 9). The age range was 30–92 years. Eight (*n* = 7) studies (37%) used a cross-sectional design, 10 (or 53%) used a prospective cohort design, 1 study (5%) used a nested matched case–control design, and 1 (5%) used a randomized intervention trial (Figure 4). The follow-up length for the prospective cohort studies ranged from 5–40 years (5, 8). The following sections present relevant study findings by nutrients and Table 1 provides a detailed summary of all included studies.

**Table 1:** Summary of all included studies (*n* = 19).

Authors, Year of Publication and Country where Study was Conducted	Study aim and research design	Sample size and population age	Validity and reliability of dietary questionnaires	How outcomes were measured	Key findings	Gaps in research
Jung, Kim, and Park, 2018 Korea (1)	Cross-sectional study to evaluate the correlation between dietary nutrient intake and glaucoma	37,982 participants who were 40 years or older	No mention of validity or reliability of 24-h recall method	Glaucomatous optic disc referred to any of the following: horizontal or vertical CDR of ≥0.5, appearance of optic disc hemorrhage, presence of RNFL defect, or violation of the ISNT rule. • Glaucoma diagnosis was made using modified ISGEO criteria.	Dietary nutrient intake was associated with open-angle glaucoma independent of IOP	The study was cross-sectional and so could not determine cause and effect relationship for dietary nutrients and glaucoma Serum analysis of nutrients was not conducted to investigate the direct association between glaucoma and nutrients.
Kang et al., 2004 USA (2)	Prospective cohort study (10–16 years) to examine dietary fat consumption in relation to POAG	Women (*n* = 76,199) and men (*n* = 40,306), 40 years or older	Validated FFQs were used	Self-reported POAG cases during follow-up, confirmed by medical chart review	Positive association between high ratio of n-3 to n-6 polyunsaturated fat and risk of POAG	No repeated eye exams in the cohorts
Yoserizal et al., 2019, Japan (3)	Cross-sectional study to investigate possible associations between nutrient intake and glaucoma in subjects of Japanese descent living in Los Angeles, CA	581 Japanese American participants in Los Angeles with a mean age of 63 years	No mention of validity or reliability of FFQ method	Fundus photography to measure CDR and rim width, to screen for glaucomatous optic disc appearances	High iron intake and low vitamin A and vegetable fat intake appeared to be associated with increased risk of glaucoma	Limited number of participants diagnosed with glaucoma. No other glaucoma examinations were carried out apart from fundus photography The participants may not have correctly reported their actual dietary intake The study was cross-sectional and so it was impossible to determine a causal relationship between diet and glaucoma
Kinouchi et al., 2018 Japan (4)	Cross-sectional study to identify lifestyle risk factors for OAG in a Japanese population	1,583 participants who were 40 years or older	No mention of validity or reliability dietary assessment tool	Fundus photographs obtained for OAG screening	Higher weekly consumption of meat was negatively associated with OAG	Some participants were not randomly selected The study did not assess all previously reported OAG risk factors, such as myopia and family history
Vergroesen et al., 2023 The Netherlands, (5)	Prospective cohort and nested matched case–control study to assess the association between the MIND diet and iOAG (since 1991, with intervals of 5 years)	8,679 participants who were 45 years or older	Validated quantitative FFQs were used	Glaucomatous VF loss in at least one eye with open ACA and no history or signs of secondary glaucoma	Greater adherence to the MIND diet was associated with decreased incidence of OAG	Small sample size and insufficient statistical power for more detailed sub-group analyses (e.g., smokers vs. non-smokers). Data on dietary patterns that were collected at baseline did not per definition reflect long term intake
Carbone et al., 2021 USA (7)	Prospective cohort study to determine if there is an association between vitamin D intake and incident glaucoma	143,389 postmenopausal women aged 50–79 years	A validated FFQ was used	Incident glaucoma defined as self-report of glaucoma development	Dietary vitamin D intake, supplements and serum levels were not significantly related to the risk of developing glaucoma	The study defined glaucoma by self-report and did not distinguish specific types of glaucoma. Serum 25 (OH) D levels were not measured in all the women and the methodology for these measurements differed.
Hanyuda et al., 2020 USA (8)	Prospective cohort study (16–40 years) to assess the long-term association between low-carbohydrate dietary patterns and incident POAG	185,638 participants with a mean age of 65.6 years	Validated FFQs were used	Incident POAG showing open ACA; no signs of secondary glaucoma; and VF defects consistent with POAG	No associations between low-carbohydrate-diet scores and POAG: However, vegetable score showed a suggestive inverse association with early paracentral VF loss	The researchers did not measure serum ketogenesis, although people who followed low-carbohydrate dietary patterns over several decades likely had higher average levels of ketone bodies.
Mylona et al., 2020 Greece (9)	Cross-sectional study to determine whether dietary practices correlate with POAG, after controlling for important risk factors, namely heredity and cardiovascular risk factors	Two samples of equal sizes (*N* = 100), with participants aged 45 years or more	FFQ validated for the EPIC-Norfolk population	Two samples of equal sizes (*N* = 100) were randomly selected from glaucoma outpatient services with and without POAG	Drinking pure fruit juice, consuming more meat with less visible fat cooked to a lower effect and modest salt consumption during cooking were seen to be practical and easy-to-observe dietary advice for any patients at risk or already suffering from POAG	Relatively small sample size needing replication to ascertain validity of the findings.
Moreno-Montañés et al., 2022 Spain (10)	Prospective cohort study (10 years) to assess associations between carbohydrates (CH) intake and glaucoma incidence	18,247 volunteer participants who were 38 years or more	A validated FFQ was used	Self-reported diagnosis of glaucoma collected at baseline and on biennial follow-up with questionnaires	High intake of total CH was associated with high risk of incident glaucoma. This association did not seem to be confounded or modified by diabetic status	Self-reported diagnosis of glaucoma was collected without ophthalmologic examination Carbohydrates intake was assessed only at baseline Other risk factors for glaucoma were not assessed
Arcelus et al., 2014 Spain (11)	Prospective cohort study (8 years) to assess associations between intake of omega 3, omega 6 fatty acids and their ratio and the incidence of glaucoma	17,128 participants initially free of glaucoma who were 40 years or more at baseline	A validated SFFQ was used	Information of new diagnosis of glaucoma in biennial follow-up questionnaires	The data suggested an association between omega 3:6 ratio intake and incident glaucoma. No significant association was observed for omega 3 or omega 6 intake and risk of glaucoma	Some degrees of misclassification were likely to exist because of the self-reported nature of exposure and outcome No information was taken on family history of glaucoma, which is an important risk factor for glaucoma
Giaconi et al., 2012 USA, (12)	Cross-sectional study to explore associations between consumption of fruits and vegetables and presence of glaucoma in older African American women	662 African American participants in the Study of Osteoporotic Fractures who were 65 years or more	A validated, self-administered block FFQ was used	Optic disc photographs and suprathreshold visual fields	Increased intake of certain fruits and vegetables high in vitamins A and C and carotenoids were associated with a decreased likelihood of glaucoma in the older African American women	Randomized controlled trials were required to determine whether intake of specific nutrients changes the risk of glaucoma
Coleman et al., 2008, USA (13)	Cross-sectional study to explore associations between the consumption of fruits and vegetables and presence of glaucoma	1,155 women, aged 65 years or older	A validated block FFQ was used	Optic nerve head photographs and 76-point suprathreshold screening visual fields	High intake of certain fruits and vegetables were associated with a decreased risk of glaucoma	Causal relationship could not be assessed
Kang, et al., 2016 USA (14)	Prospective cohort study (26–28 years) to evaluate the association between POAG and dietary nitrate intake derived mainly from green leafy vegetables	63,893 women and 41,094 men, who were 40 years or older	Validated SFFQs were used	Incident POAG confirmed with medical records defined by IOP (≥22 or < 22 mm Hg) or by glaucomatous VF loss pattern at diagnosis	Higher dietary nitrate and green leafy vegetable intake were associated with low POAG risk, particularly POAG with early paracentral VF loss at diagnosis	The study was observational There might have been a possibility of residual confounding by other dietary factors for example, nitrate-rich vegetables may have had other nutrients, though adjustments were made for intake of other nutrients
Mehta et al., 2023 USA (15)	Randomized intervention trial to test if dietary modification (DM) (a diet low in fat, with increased vegetables, fruits, and grains) alters the risk for incident POAG (Mean follow-up, 12 years; mean DM duration, 5 years)	Randomized DM intervention group = 9,340; control group = 13,877, with a mean age of 64 years	No mention of validity or reliability of the FFQ used	POAG was defined as first claim with the International Classification of Diseases, Ninth or Tenth Revision, codes	DM did not reduce incident POAG. DM in participants in the lowest quartile group for percentage calories from total fat at baseline increased the risk of incident OAG among women regardless of age or race	Billing codes were used as an as an end point in identifying POAG, and data on ocular risk factors were not included in the study
Kang et al., 2014 USA (16)	Prospective cohort study (24–30 years) to assess the relationship between intake of folate, vitamin B6 and vitamin B12 and risk of exfoliation glaucoma or glaucoma suspect (EG/EGS)	120,201 participants who were 40 years or older	Validated SFFQs were used	Incident EG/SEG based on IOP exceeding 21 mm Hg, CDR of 0.6 or higher, or visual field loss consistent with glaucoma in the eye with exfoliation material	Higher total folate intake was associated with lower risk of EG/EGS	In-person eye examinations were not conducted but EG/SEG cases were identified by self-report and then confirmed by medical records. Higher B vitamin intake may have been a marker of another unmeasured risk factor and may have caused some residual confounding
Ramdas et al., 2012 The Netherlands (17)	Prospective cohort study (10 years) to determine whether the dietary intake of nutrients that either have anti-oxidative properties or influence blood flow are associated with incident OAG	3,502 participants who were 55 years or older	Validated SFFQs were used	Incident glaucoma with measurements of the intraocular pressure and perimetry	Low intake of retinol equivalents and vitamin B1, and a high intake of magnesium appeared to be associated with an increased risk of OAG	Limited number of participants who developed OAG during follow-up
Kang et al., 2003 USA (18)	Prospective cohort study (10–16 yrs) to examine the relationship between dietary antioxidant intake and primary POAG risk	116,484 participants aged over 40 years	Validated SFFQs were used	Glaucoma cases were confirmed by medical chart review to have POAG with visual field loss	There were no strong associations between antioxidant consumption and risk of POAG	There might have been a possibility of confounding by other variables, such as family history of POAG
Vergroesen et al., 2023 The Netherlands (19)	Prospective cohort study (1991 onwards, every 4–5 years) to assess whether the inflammatory potential of diet affects iOAG	7,436 participants free of glaucoma at baseline who were greater than 45 years of age.	Validated FFQs were used	iOAG defined as glaucomatous visual field loss with reproducibility of the defect	While a high dietary inflammatory index was significantly associated with increased inflammation it was not associated with OAG	Although multiple confounders were adjusted for, it was possible that additional confounding factors may have influenced the results including genetic predisposition for glaucoma, use of specific medications, and other (lifestyle) factors
Wierzbowska et al., 2008 Poland, (20)	Cross-sectional study to assess the differences between the prevalence of risk factors in patients with age-related macular degeneration (AMD), glaucoma and both diseases	Glaucoma group (34 patients), AMD group (83 patients). 30–92 years	No mention of validity or reliability dietary assessment tool	Presence of typical visual field defects with Humphrey perimetry; and ophthalmoscopic examination of the optic disc (for CD ratio and presence of hemorrhages)	High fat intake in diet was lower in the Glaucoma Group, compared to the AMD Group (OR 0.5 *p* = 0.03)	The obtained results needed confirmation on a larger scale

ACA, Anterior chamber angle; AMD, Age-related macular degeneration; CDR, cup-to-disc ratio; DM, dietary modification; EG/EGS, exfoliation glaucoma or glaucoma suspect; FFQ, Food Frequency Questionnaire; iOAG, incident open-angle glaucoma; ISNT rule, neuroretinal rim thickness in the order of inferior > superior > nasal > temporal; OAG, Open-angle glaucoma; RNFL, Retinal nerve fiber layer defect; POAG, Primary open-angle glaucoma; SFFQ, Semi-quantitative Food Frequency Questionnaire; vCDR, vertical cup to disc ratio; VF, visual field; and WHI, Women Health Institute.

### 3.1 Carbohydrates

Two prospective cohort studies assessed associations between intake of carbohydrates and incidence of glaucoma (8, 10). One study observed that participants in the highest quartile of carbohydrates intake at baseline had significantly higher risk of glaucoma as compared to participants in the lowest quartile [HR 1.50 (95% Confidence interval (CI): 1.01–2.25), *p* for trend = 0.042]. This association did not seem to be confounded or modified by diabetic status (11). The second prospective cohort study did not find any associations between 3 types of low-carbohydrate-diet scores (overall score, animal score and vegetable score) and POAG. However, the vegetable score in that research was indicative of an inverse association with early paracentral visual field (VF) loss (highest vs. lowest decile MVRR = 0.78 [95% CI, 0.55–1.10]; *P*_trend_ = 0.12). The authors therefore concluded that substituting high consumption of fat and protein from vegetable sources for carbohydrates was associated with a lower risk of a POAG subtype, with initial paracentral VF loss.

### 3.2 Proteins

Three observational studies examined associations between intake of animal protein and glaucoma risk. Kinouchi et al., observed that a high frequency of meat consumption appeared to be associated with reduced risk of open-angle glaucoma (OAG) in a cross-sectional study in Japanese women (4). The number of days per week that participants consumed meat (mean ± SD; OAG: 1.7 ± 1.2 days, non-OAG: 2.7 ± 1.5 days) was negatively associated with OAG (OR = 0.61; 95% CI: 0.43–0.88; *p =* 0.007) (4). Likewise, Mylona et al., in another cross-sectional analysis, concluded that consuming more meat was a useful advice for patients at risk of or already suffering from POAG when they observed that one difference between POAG patients and nonglaucoma subjects was that glaucoma patients eat less meat than nonglaucoma subjects (9).

Again, including fish in one Mediterranean-DASH Intervention for Neurodegenerative delay (The Mind) Diet appeared to be a protective factor for OAG in a prospective cohort study and nested matched case-control analysis (5). Greater adherence to the diet was associated with decreased incident OAG risk (odds ratio [95% confidence interval]: 0.80 [0.66 to 0.96], for each 10-percent increase in adherence) (5).

### 3.3 Fats

Five investigations assessed fat intake and risk of glaucoma. Two prospective cohort studies observed positive associations between high omega 3:6 ratio and glaucoma risk in RR = 1.49 (1.11, 2.01); (hazard ratio (HR): 1.91 [95%CI: 1.05–3.46], p for trend 0.03) (2, 12); but no significant association was observed for either omega 3 or omega 6 intake. One cross-sectional study observed that low vegetable fat intake was associated with increased risk of glaucoma (OR: 0.957, *p* = 0.004) in Japanese Americans after multivariate logistic regression analysis (3). Another cross-sectional study observed that dietary intake of visible fat was higher in glaucoma patients than in patients without glaucoma and considered consuming less fat to be good dietary advice for patients at risk of or already suffering from POAG (9).

### 3.4 Fruits and vegetables

Five investigations assessed the relationship between glaucoma risk and consumption of fruits and/or vegetables. Two cross-sectional studies observed that high intake of certain fruits and vegetables were associated with decreased risks of glaucoma (13, 14). In one of them, the odds of glaucoma risk were decreased by 69% (odds ratio [OR], 0.31; 95% confidence interval [CI], 0.11 to 0.91) in women who consumed at least one serving per month of green collards and kale compared with those who consumed fewer than one serving per month; by 64% (OR, 0.36; 95% CI, 0.17 to 0.77) in women who consumed more than two servings per week of carrots compared with those who consumed fewer than one serving per week; and by 47% (OR, 0.53; 95% CI, 0.29 to 0.97) in women who consumed at least one serving per week of canned or dried peaches compared with those who consumed fewer than one serving per month (14). In the second study, African American women who ate 3 or more servings/day of fruits/fruit juices were 79% (odds ratio [OR] = 0.21; 95% confidence interval [CI]: 0.08–0.60) less likely to have glaucoma than women who ate less than one serving/day. Women who consumed more than 2 servings/week of fresh oranges (OR = 0.18; 95%CI: 0.06–0.51) and peaches (OR = 0.30; 95% CI: 0.13–0.67) had decreased odds of glaucoma compared to those consuming less than one serving/week. For vegetables, >1 serving/week compared to ≤1 serving/month of collard-greens/kale decreased the odds of glaucoma by 57% (OR = 0.43; 95%CI: 0.21–0.85). There was also a protective trend against glaucoma in those consuming more fruit/fruit juices (*p* = 0.023), fresh oranges (*p* = 0.002), fresh peaches (*p* = 0.002), and collard greens/kale (*p* = 0.014). Higher consumption of carrots (*p* = 0.061) and spinach (*p* = 0.094) also showed some associations (13).

In the same vein, another cross-sectional study concluded that drinking pure fruit juice was one beneficial dietary advice for patients at risk or already suffering from POAG when it found that one difference between clinical cases of POAG and controls was that glaucoma cases drank less pure fruit juice than controls (9).

Also, high intake of green leafy vegetable was associated with a lower POAG risk, particularly POAG with early paracentral VF loss at diagnosis in a prospective cohort study (15). Compared with consuming 0.31 servings/day, the multivariable rate ratio (MVRR) for consuming 1.45+ servings/day was 0.82 for all POAG (95%CI, 0.69, 0.97; p-trend = 0.02) and 0.52 for POAG with paracentral VF loss (95%CI, 0.29, 0.96; p-trend = 0.0002) (15). Similarly, high intake of green leafy vegetables, and berries seemed to be protective factors against open-angle glaucoma in a nested matched case–control study (5).

For all that, a randomized intervention trial that assessed whether dietary modification (such as a diet low in fat, but high in vegetables, fruits, and grains) could alter the risk for incident primary open-angle glaucoma (POAG), found no overall benefit of dietary modification (HR, 1.04; 95% CI, 0.96–1.12) (16).

### 3.5 Vitamins and provitamins

Five observational studies examined associations between intake of vitamins/provitamins and glaucoma risk. One cross-sectional study found protective trends with high intakes of vitamin A (*p* = 0.011), vitamin C (*p* = 0.018), and *α*-carotene (*p* = 0.021), and close to statistically significant trends with *β*-carotene (*p* = 0.052), folate (*p* = 0.056), and lutein/zeaxanthin (*p* = 0.077) in older African American women (13). Another cross-sectional study observed that low intake of vitamin A was associated with increased risk of glaucoma (OR: 0.365, *p* = 0.019) (3). Some other prospective cohort study showed a suggestive trend of reduced glaucoma risk with higher intake of folate (vitamin B-9) i.e., compared with the lowest quintile of cumulatively averaged total folate intake, the MVRR of exfoliation glaucoma or glaucoma suspect for the highest quintile (Q5; ≥ 654 μg/day) was 0.75 (95% Confidence Interval [CI]: 0.54–1.04; p for linear trend = 0.02) (17). Low intake of retinol equivalents and vitamin B1 also appeared to be associated with an increased risk of OAG in another prospective cohort study. The hazard ratio for retinol equivalents (highest versus lowest tertile) was 0.45 (95% confidence interval 0.23–0.90), and for vitamin B1 0.50 (0.25–0.98) (18).

Conversely, high intake of dietary niacin (vitamin B3) was associated with glaucoma risk in a cross-sectional study (*p* = 0.013) OR (95% CI) (1). An additional prospective cohort study observed no associations between glaucoma risk and consumption of either *α*-carotene, *β*-carotene, β–cryptoxanthin, lycopene, lutein/zeaxanthin, vitamin A, vitamin C or vitamin E (11). Also, Carbone et al., did not find any associations between incident glaucoma and dietary vitamin D intake, supplements or serum levels in another prospective cohort study in postmenopausal women (7).

### 3.6 Minerals

Three observational studies examined associations between intake of minerals and glaucoma risks. A prospective cohort study observed that high dietary nitrate intake was associated with lower POAG risk, particularly POAG with early paracentral VF loss at diagnosis (15). Green leafy vegetables accounted for 56.7% of nitrate intake variation. Compared with consuming 0.31 servings per day, the MVRR for consuming 1.45 or more servings per day was 0.82 for all POAG (95% CI, 0.69–0.97; P for trend = 0.02) and 0.52 for POAG with paracentral VF loss (95% CI, 0.29–0.96; P for trend <0.001).

Alternatively, high iron intake appeared to be associated with increased risk of glaucoma in Japanese descent living in the Los Angeles populations (odds ratio [OR]: 1.303, *p* = 0.004) in a cross-sectional study; and high magnesium intake appeared to be associated with increased risk of OAG (HR = 2.25 [1.16–4.38]) in one prospective cohort study (3, 18).

### 3.7 Miscellaneous food

Dietary patterns with high inflammatory potential were not associated with OAG in some other prospective cohort study (OR [95% CI]: 1.09 [0.95–1.24] per point) (P trend = 0.68) (19); and modest salt consumption during cooking was perceived to be a helpful dietary advice for patients at risk or already suffering from POAG after a cross-sectional study observed that clinical cases of glaucoma differed from controls with respect to modest salt consumption during cooking i.e., people with POAG seemed to consume more salt than those without glaucoma (9).

## 4 Discussion

The objective of this review was to determine the breadth of evidence on “diet and glaucoma” and evaluate the extent and nature of research that has been undertaken in this area to determine any existing gaps in knowledge. About 80% of the studies identified by this review found some significant associations between glaucoma and dietary intake. High dietary intake of vegetables (like carrots, kale and green collards) and fruits (like oranges, berries, and peaches), all rich in vitamins A and C and carotenoids, seemed to be protective factors for glaucoma (5, 13, 14). Additionally, higher consumption of meat, fish, vegetable fat, and high intake of nutrients such as dietary nitrate, retinol equivalents, folate, vitamin A, and vitamin B1 were associated with lower risk of glaucoma (3–5, 8, 13, 15–17). Conversely, high intake of carbohydrates, fats, salt, niacin, iron, magnesium, and high omega 3:6 ratio (n-3 to n-6 polyunsaturated fat) were associated with increased risk of open-angle glaucoma (1–3, 9, 12, 18, 20). These findings suggest that dietary intake may be another modifiable factor in glaucoma, apart from intraocular pressure. Furthermore, dietary changes may be of importance to people with glaucoma (21–23).

However, majority (95%) of research designs in this scoping review were fundamentally observational, i.e., 37% were cross-sectional, 53% used a prospective cohort design, and 5% used a nested matched case–control. Only 1 study (or 5%) used a randomized interventional trial. This is probably why dietary modification is not usually included in routine clinical management of glaucoma, since most data are from observational studies (15, 23). Several limitations are normally considered when interpreting results from observational studies even if such studies simulate “real world” settings (24). One limitation is that they do not usually establish causal relationships (3, 14). Furthermore, they conventionally lack temporality, and, in some contexts, it can be challenging to identify whether dietary intake truly preceded the glaucoma outcome. For example, a cross-sectional study by Coleman *et al*, in 2008, could not establish a causal relationship because the information on dietary intake and the presence of glaucoma were obtained during the same visit (14). More experimental designs, especially randomized controlled trials, which are the ‘gold standard’ for clinical research and assessing effectiveness of therapy, are therefore required to determine specific dietary patterns that are beneficial (1, 25, 26). These experimental techniques can improve our understanding of the implications of dietary intake in glaucoma because they are designed to answer very specific questions about a particular treatment strategy and can establish evidence of causation between both variables (25, 27).

In addition, while almost all the studies in this review investigated dietary intake with validated food frequency questionnaires and assessed potential confounders, only two studies (or 11%) went further to include the use of nutritional biomarkers (7, 19). The use of only subjective dietary tools to assess dietary intake in these studies is a major limitation because the subjective tools depend solely on self-reporting and are vulnerable to systematic bias, influenced by factors such as age, gender, social appeal and approval, and thus present challenges to obtaining accurate dietary intake (28, 29). One reason is that people rarely perceive what they eat and how much, and may not correctly report their actual dietary intake (1, 3, 30). Individuals are not always able to remember all foods consumed or the specific components of the food (e.g., condiments in hamburgers) and have difficulty establishing precise portion sizes and sometimes underestimate dietary intake (29). This shortcoming can be overcome using dietary biomarkers, which are able to objectively assess dietary consumption (or exposure) without the bias of self-reported dietary intake errors (29). They are desirable for their ability to more accurately assess nutritional intake/status, and more meticulously connect dietary intake with glaucoma risk. The biomarkers are also better indicators of dietary intake in cases where nutrients and food components vary considerably for the same food depending on the place and way the food was cultivated, or how it was refined (31). The use of such biomarkers along with dietary questionnaires could help us gain a better understanding of the relationship between glaucoma and dietary intake (1).

## 5 Conclusion

Several studies identified by this review found some significant associations between glaucoma and dietary intake. However, most data were observational reporting only associations. Furthermore, most investigations used only subjective dietary questionnaires to assess dietary intake. Well-designed studies including randomized controlled trials that can prove causality are therefore required in this area of scholarship. Improvements in the quality and quantity of research will create an evidence base to support policy and interventional efforts for moderating the negative blinding effects of glaucoma.
